# Novel partially cross-linked nanoparticles graft co-polymer as pore former for polyethersulfone membranes for dyes removal

**DOI:** 10.1016/j.heliyon.2023.e21958

**Published:** 2023-11-08

**Authors:** Kadhum M. Shabeeb, Wallaa A. Noori, Ali A. Abdulridha, Hasan Sh Majdi, Mohammad N. Al-Baiati, Ali A. Yahya, Khalid T. Rashid, Zoltán Németh, Klara Hernadi, Qusay F. Alsalhy

**Affiliations:** a Department of Materials Engineering, University of Technology- Iraq, Alsinaa Street 52, 10066 Baghdad, Iraq; b Membrane Technology Research Unit, Chemical Engineering Department, University of Technology- Iraq, Alsinaa Street 52, 10066 Baghdad, Iraq; c Department of Pharmacy, Alsafwa University College, Karbala, Iraq; d Department of Chemical Engineering and Petroleum Industries, Al-Mustaqbal University, Babylon, 51001, Iraq; e Department of Chemistry, College of Education for Pure Sciences, University of Kerbala, 56001, Kerbala, Iraq; f Advanced Materials and Intelligent Technologies Higher Education and Industrial Cooperation Centre, University of Miskolc, H-3515, Miskolc, Hungary

**Keywords:** Nanoparticles, Graft co-polymer, Textile, Dye removal, Ultrafiltration, Membrane fouling

## Abstract

A newly developed water-soluble polymeric nano-additive termed “partially cross-linked nanoparticles graft copolymer (PCLNPG)" has been successfully synthesized and harnessed as a pore former for modifying a polyethersulfone ultrafiltration membrane for dyes removal. The PCLNPG content was varied in the PES polymeric matrix aiming to scrutinize its impact on membrane surface characteristics, morphological structure, and overall performance. Proposed interaction mechanism between methylene blue (MB), methyle orange (MO), and malachite green (MG) dyes with PES membrane was presented as well. Hydrophilicity and porosity of the novel membrane increased by 18 and 17 %, respectively, when manufactured with a 3 Wt. % PCLNPG, according to the findings. Besides this, the disclosed increased porosity, rather than the hydrophilic properties of the water-soluble PCLNPG, was the principal cause of the diminished contact angle. Meanwhile, raising the PCLNPG content in the prepared membrane made worthy shifts in its structure. A sponge-like region was materialized near the bottom surface as well. The membrane's pure water flux (PWF) synthesized with 3 Wt.% PCLNPG recorded 628 LMH, which is estimated 3.95 fold the pristine membrane. MG, MB, and MO dyes were rejected by 90.6, 96.3, and 97.87 %, respectively. These findings showed that the performance characteristics of the PES/PCLNPG membrane make it a potentially advantageous option to treat the textile wastewater.

## 1 Introduction

Textile industries are among the industries that lead to the release of humongous amounts of water contaminated with synthetic dyes [1,2]. The unacceptable concentrations of dyes disrupt aquatic plant photosynthetic processes by preventing sunlight penetration [3]. Besides, human exposure to these pollutants for a long time leads to serious health problems [4,5]. MO, MB, and MG dyes are commonly used in textiles and pharmaceuticals industry [[6], [7], [8]]. Due to the sheer complexity of these dyes' structures, conventional methods of removing them from wastewater seem to be difficult. Among the available broad-spectrum wastewater treatment methods, membrane-based separation processes stand out as a practical solution to problems caused by water contamination. Membrane processes are characterized by their high efficiency, ease of fabrication, and low cost [9]. In this context, the high water permeation, effective pollutant removal, and low energy requirements made ultrafiltration (UF) membrane as an attractive option in different applications, such as the pharmaceutical, food, and wastewater treatment industries [10]. The major challenge for UF applications, however, is membrane fouling induced by the inherent hydrophobic nature of organic polymers. As a consequence, membrane fouling diminishes pure water production in industrial operations.

Surface membrane modifications that improve surface hydrophilicity and morphology are effective methods for addressing membrane fouling issues. This could be accomplished by revising the membrane surface characteristics to achieve novel membrane characteristics. To meet this target, it has been reported that the impregnation of hydrophilic materials is an excellent way to improve these properties [11,12]. Nonetheless, depending on the membrane process design required, researchers have proposed a plethora of additives and surface modification techniques [[13], [14], [15], [16]]. The integration of hydrophilic nanomaterials is one of these exceptional methods. Due to the outstanding properties of these nanoscale additives, this field has grown in popularity. In comparison to their bulk size, nanomaterials could bestow desired features on membranes. Among these anticipated features are preferable structural changes and enhanced hydrophilicity. Dimensions (e.g., 0D, 2D, and 3D), shape (e.g., nanoparticles, nanorods, nanotubes, nanosheets … etc.), materials (carbon, metal/metal oxide, cellulose, polymers … etc.), and so on could all be used to categorize nanomaterials. The membrane stability is determined by how these nanomaterials interact with the polymeric matrix. Nanomaterials have weak interactions with organic polymers, depending on how they are synthesized, and may eventually leach out of the membrane structure. However, the use of such nanomaterials may harm the environment due to the leakage of these materials from membranes, resulting in membrane damage and decreased performance.

Besides that, utilising the pore former additives during the membrane fabrication process is an appealing method to improve the membrane surface features [17]. Once pore former agents are impregnated into the polymer casting solution, significant structural changes occur, likely to result in membranes with high porosity and pore density when compared to pristine membranes. Moreover, polymeric additives used as pore formers have been displayed to improve the hydrophilicity of the membrane surface [[17], [18], [19], [20], [21], [22]]. It is worthy to mention that there are many studies that have dealt with the simulation of ultrafiltration membrane formation [[23], [24], [25], [26]].

In this work, novel water-soluble polymeric nanoadditives called “partially cross-linked nanoparticles graft copolymer (PCLNPG)” was prepared and utilized as pore former for PES membrane modification. The PCLNPG loading content into the PES membrane matrix was manipulated to probe their impact on the structure and performance of the membrane. Set of characterization techniques were employed to determine these new features. This includes; SEM, FTIR, porosity and contact angle. PWF and the rejection of MG, MB and MO dyes were determined to evaluate the membrane performance.

## 2 Experimental method

### 2.1 Chemicals

Fom BASF company (Germany), PES polymer (MW = 51 kDa) was purchased and employed as the host polymer. Dimethylacetamide (DMAc) used for casting solution preparation, phthalic anhydride, glycerol, dimethyl sulfoxide (DMSO) and p-Xylene employed for preparation of PCLNPG were purchased from Sigma-Aldrich, Germany. Methylene blue dye (Mw = 319.85 g/mol), methyl orange dye (Mw = 269.31 g/mol) and malachite green dye (Mw = 364.92 g/mol) used for membrane rejection test were purchased from Nanjing Duly Biotech Co., China.

### 2.2 Preparation of PCLNPG

PCLNPG was made by dissolving Phthalic Anhydride (740.5 g) in DMSO (70 ml) at 110 °C until the solution became clear, then add 184 g of glycerol. To separate the water molecules produced during the reaction, p-xylene (15 ml) was gradually added. The suspension solution was then created by adding cold deionized water. Finally, filtration was used to separate the PCLNPG formed.

**Figure:**
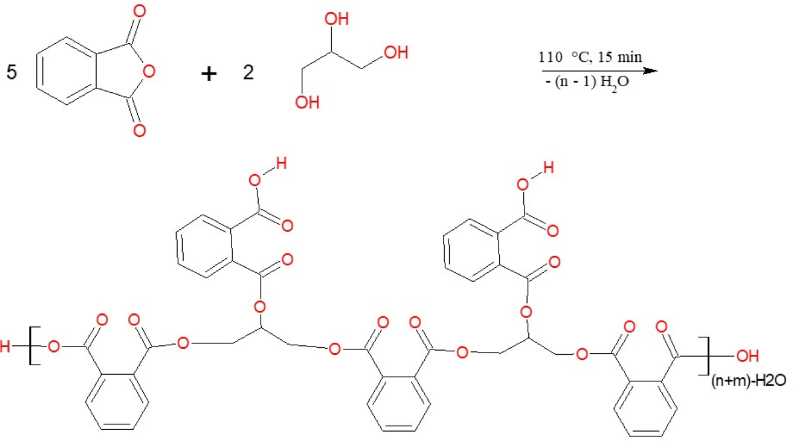

Then, drying for 2 h at 80 °C was used to produce PCLNPG powder. The reaction described refers to the formation of PCLNPG.

### 2.3 PCLNPG characterization

Nuclear magnetic resonance NMR is utilized to identify the prepared PCLNPG. ^13^C NMR (at 101 MHz) and ^1^H NMR (at 400 MHz) spectra were recorded by a Bruker DPX-300 spectrometer, Germany. The size of PCLNPG powder was determined by atomic force microscope (AFM).

### 2.4 Preparation of membranes

PES membrane was prepared via classical phase inversion method. Initially, a range of PCLNPG content (see composition in Table 1) was individually dissolved in DMAc for 1 h. Then, fixed amount of 20 % PES was gradually droped to the solution and mixed with a magnetic stirrer. Blending continue once the casting solution turns into clear, yellowish and homogeneous. An ultrasonic bath has been employed to expel the bubbles formed during preparation. An automatic casting machine with 200 μm clearance gap was used for membrane casting. To finalize the coagulation process, the casted layer was immediately submerged in pure water basin at 40 °C. The prepared membrane was then taken out of the bath, rinsed repeatedly under current water and stored in the distilled water ready for characterization [22].

**Table 1:** PES/PCLNPG solutions composition.

Membrane	PES (wt.%)	Solvent (wt.%)	PCLNPG (wt.%)
Aᴏ	20	80	0
A1	20	79	1
A2	20	78	2
A3	20	77	3
A4	20	76	4

## 3 Membrane characterization

### 3.1 FTIR spectroscopy

Fourier Transform Infrared Spectroscopy (FTIR) (TENSOR-27, Bruker Optics Co.) with range of 500–4000 cm^−1^ was utilized to confirm the chemical compositions of the prepared membranes.

### 3.2 Morphology of membrane

Membrane morphology is characterised by its structural features and physical properties. The visualisation of membrane structure was conducted by TESCAN VEGA3 SB scanning electron microscope, manufactured by EO Elektronen-Optik-Service GmbH in Dortmund, Germany. Prior to conducting cross-sectional imaging, the prepared sheets underwent cryogenic freezing by liquid nitrogen, fracturing, and subsequent sputtering with thin gold film measuring 5 nm in thickness.

### 3.3 Measurement of contact angle (CA)

The determination of hydrophilic character of the membrane was conducted by CA measurements with the aid of an optical contact angle instrument (CAM110, Tainan, Taiwan) [27]. The membrane samples were affixed to a glass substrate through the utilisation of double-sided adhesive tape. Subsequently, a total of five droplets of deionized water, each measuring 5 μL, were strategically positioned at various locations upon the membrane's surface in order to quantify the contact angle. A minimum of five measurements were acquired for each sample, and subsequently, the obtained values were subjected to averaging.

### 3.4 Porosity and pore size

Membranes porosity was evaluated using the dry/wet method. The samples measuring 2 cm by 2 cm were excised and subjected to desiccation within an oven for a duration of 5 h at 50 °C. The weight of the membrane sample was measured. The specimen was subsequently immersed in deionized water for a duration of 1 h, subsequently extracted and gently dried using a dry tissue to eliminate any residual water droplets, and subsequently subjected to another measurement [28]. The measurement of membrane porosity *ε* was conducted utilising Equation (1). Three estimations of porosity were conducted for each membrane sample, and subsequently, the outcomes were subjected to an averaging process.

(1) $$\varepsilon \left(\%\right)=\left(1-\frac{\rho_{m}}{\rho_{p}}\right)\times 100$$

where ρ_p_ and ρ_m_ are the polymer and membrane density respectively.

Mean and distribution of PES/PCLNPG membrane pore size were estimated using Image-J version 1.50e [29].

### 3.5 Membrane performance

A laboratory-scale cross-flow mode was utilized to assess the membrane performance. The experimental setup was furnished with a membrane cell possessing an active area measuring 21.66 square centimetres. The determination PWF was conducted utilising Eq. (2) as stated in reference [30].

(2) $$J=\frac{V}{A\times t}$$

where PWF was denoted as (J), A was the effective area, and V is the volume collected of permeate watre during time (t) of the operation.

The rejection of membranes was assessed employing three types of synthetic dyes; MG, MO and MB. The feed concentration for each dye was fixed at typical concentration (30 ppm) [31]. pH of the dye feed solutions was maintained to 7 by diluted solutions of NaOH and HCl. Both feed and permeate samples were tested using UV spectroscopy where the maximum wavelength for each dye (λ max) was 665, 618 and 464 nm for MB, MG and MO, respectively. Equation (3) [32] was utilized to analyze the dye solution rejection.

(3) $$dye\;rejection\%=\left(1-\frac{C_{f}}{C_{0}}\right)\times 100$$

where C_f_ and C_0_ are the final and initial concentrations of dyes in ppm.

## 4 Results and discussion

### 4.1 PCLNPG identification

**Fig. 1:**
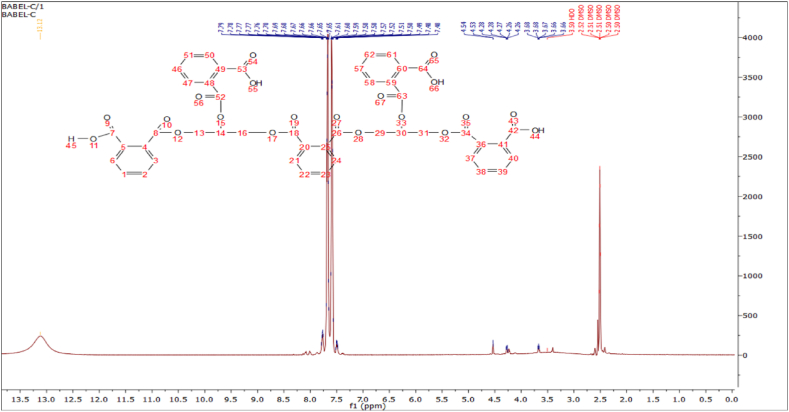
^1^H NMR of the PCLNPG additive.

**Fig. 2:**
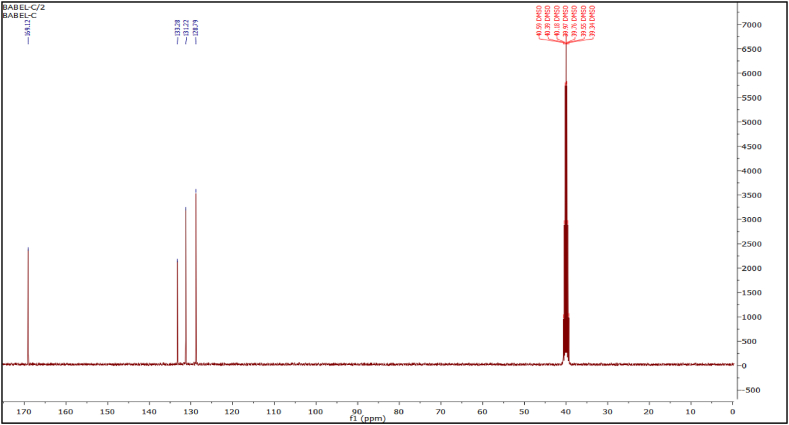
^13^C NMR of PCLNPG additive.

Fig. 1 depicts the ^1^H NMR spectra of PCLNPG. The singlet at (13.12 ppm) refers to the terminal acidic hydroxyl groups, the multiples at 7.79–7.48 ppm to the aromatic protons of benzene rings, the doublet at 4.45 and 4.28 ppm to the protons of the CH_2_ groups, and the quintet at 3.68–3.66 ppm to the protons of the CH groups. The ^13^C NMR spectra of PCLNPG are depicted in Fig. 2. The chemical shifts δ are as follows: 169.12 ppm (for the carbon of the carboxylic group 7 & 42), 136.72 ppm (carbon 8, 34, 18 & 26), 136.64 ppm (carbon of esteric carbonyl groups 2 & 38), 133.28 ppm (aromatic carbons 1, 4, 24 & 39), 133.28 ppm (aromatic carbons 5, 20, 25 & 36), 131.73 (carbon 13, 16, 29 & 31).

**Fig. 3:**
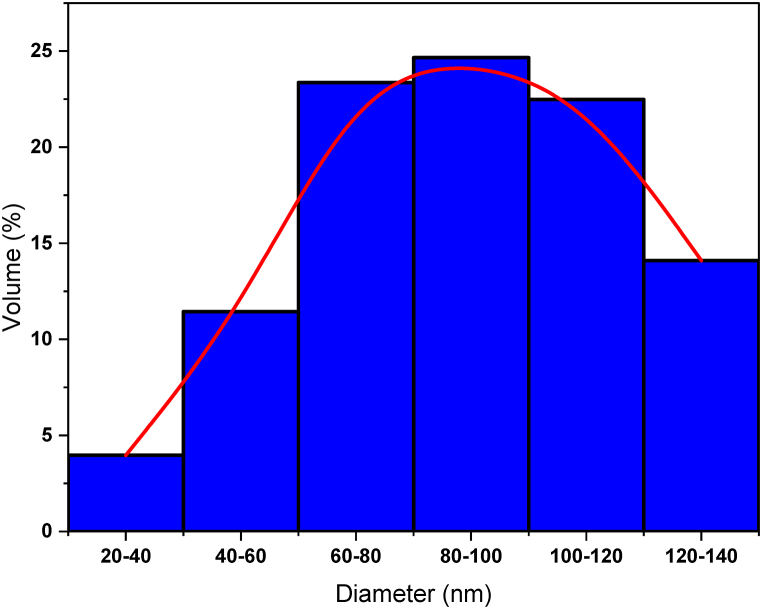
The volume percent of PCLNPG particle size distribution.

Meanwhile, the volume percent of produced PCLNPG was depicted in Fig. 3. As shown, obtained diameters were ranging from 20 to 140 nm. According to the findings, the molecular mean size of the polymeric nanoparticles was about 77.99 nm.

### 4.2 FTIR spectroscopy

**Fig. 4:**
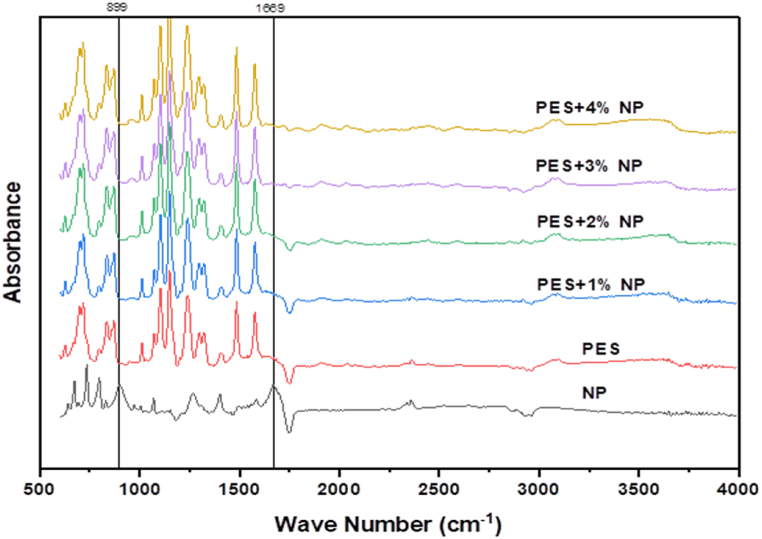
FTIR of PCLNPG additive and PES/PCLNPG membranes.

**Fig. 5:**
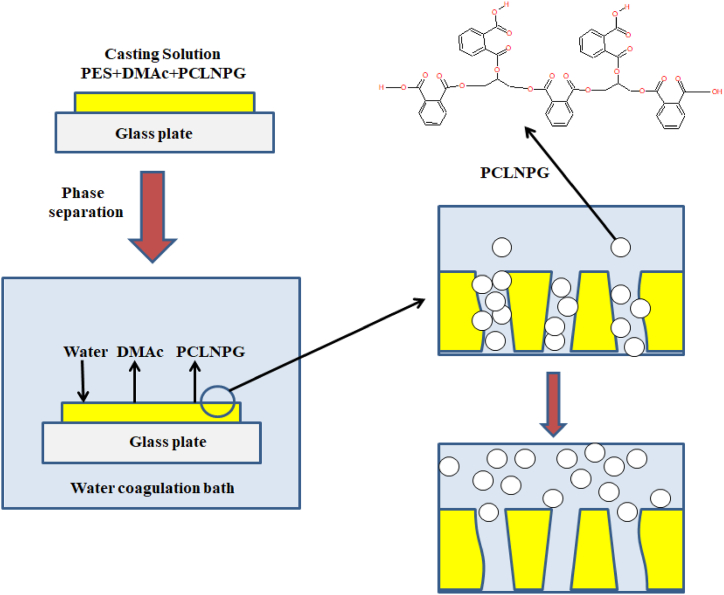
Mechanism of pore formation during the preparation of membrane.

Fig. 4 displays the spectra of Fourier Transform Infrared of both the neat and modified Polyethersulfone membrane, which has been subjected to varying concentrations of PCLNPG nano-polymer. The distinctive spectral features of PES membranes, both with and without the inclusion of PCLNPG additives, were determined by the presence of specific absorption peaks. These peaks were observed at wavenumbers of 1104.6 cm-1, 1239.9 cm-1, and 1149.43 cm-1. The peak at 1104.6 cm-1 corresponds to the stretching of (C–O) bonds, while the peaks at 1239.9 cm-1 and 1149.43 cm-1 correspond to the stretching of (C–*O*–C) bonds and (O=S=O) symmetric bonds, respectively. The aforementioned findings provided further support to the conclusions drawn in the prior investigations conducted by Hosseini and Alvi [33,34]. In addition, the detected peaks at approximately 1577.7 cm-1 and 1485.9 cm-1 have been attributed to the presence of aromatic rings, whereas the peak at 3100 cm-1 has been attributed to the stretching of C–H bonds [35]. The observed spectral features at 560 cm-1 correspond to the scissoring deformation of the SO2 molecule. Further, the peaks observed at 12.94 cm-1 and 1175 cm-1 are attributed to the asymmetrical and symmetrical stretching of the SO_2_ molecule, respectively. The peak 1244 cm-1 [36] can be assigned to the Aryl-*O*-Aryl C–O stretching vibration mode. Fig. 4 demonstrates that the absorption bands observed in the spectra of the PES/PCLNPG and pristine PES membranes exhibit identical characteristics. In addition, it is noteworthy to mention that for PES/PCLNPG membrane, a conspicuous absence of the spectral peaks at 1669 and 899 cm-1 was observed. This observation suggests that the PCLNPG constituent experienced a process of hydrolytic degradation, which lead to a significant change in membrane structure through pores formation. The observed degradation phenomenon may be attributed to the high concentration of hydroxyl groups (OH-) present in the structure of nanoparticle. These hydroxyl groups facilitate the hydrogen bond to be formed between (OH-) and water molecules in the coagulation bath. Due to its inherent hydrophilic properties, the water-soluble constituent known as PCLNPG was effectively extracted from the coagulation bath. Fig. 5 depicts the postulated mechanism underlying the pore formation in the PES/PCLNPG membrane during its preparation.

### 4.3 SEM of PES/PCLNPG membrane

**Fig. 6:**
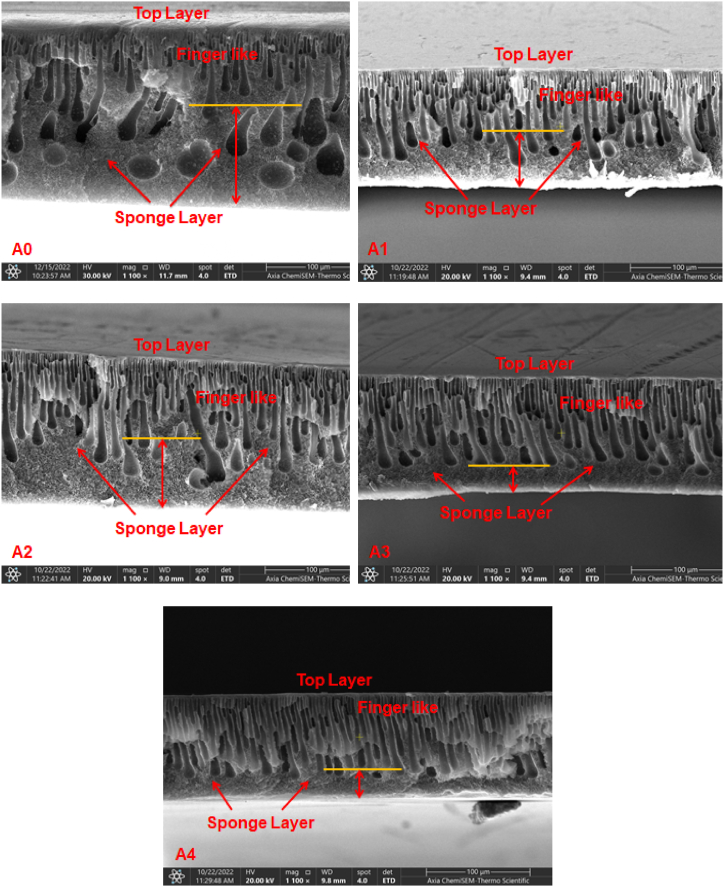
SEM images for Cross-sectional structure SEM images of pristine PES and PES/PCLNPG membranes A0, A1, A2, A3 and A4 with different wt% of PCLNPG additives of 0, 1, 2, 3, and 4 wt% respectively.

SEM was used to observe and analyze the cross-sectional structures and surface of the membranes. Fig. 6 depicts the images for cross-sectional of PES membranes that have been fabricated using different concentrations of PCLNPG. The observed entity perturbed a prototypical PES membrane configuration, characterised by a symmetrical arrangement. This configuration consists of a compact uppermost layer (referred to as the “skin layer”) that is undergirded by a permeable sublayer (known as the “support layer”). The separation phenomenon was initiated by the presence of the skin layer, which acted as a catalyst for the process. Meanwhile, the membrane mechanical integrity and membrane robustness were conferred by the support layer, ensuring its structural stability. Distinct digitiform cavities are observable within the underlying sublayer, situated immediately below the superficial topmost layer. The data also revealed that the membrane structure exhibited noticeable alterations following the involving of PCLNPG into the polymer solution. In the scenario involving an unaltered PES membrane, a multitude of macrovoids became evident within the sponge layer, as depicted in Fig. 6A0. The presence of macropores diminishes as the concentration of PCLNPG additives increases, leading to a limited sponge-like structure observed in the membrane at 3 and 4 wt% PCLNPG (as depicted in Fig. 6A3 and A4). Furthermore, it was observed that a direct correlation existed between the concentration of PCLNPG nanopolymer and the magnitudes of both size and quantity of finger-like pores. Fig. 6 illustrates the function of PCLNPG during the progression of the membrane structure. It is important to mentioning here that the dense layer thickness exhibited a slight decrease upon the involving of PCLNPG nano-polymer into the PES solution. The observed phenomenon can be ascribed to the immadiate exchange rate between dimethylacetamide (DMAc), and water during the formation of membrane. This effect becomes more pronounced with the addition of PCLNPG additives, as previously documented in our earlier research [29]. As the elongation of the digitiform apertures progressed towards the basal region of the membrane, the substantial stratum underwent removal. Fig. 6A1, A2, A3, and A4 depict the transformation of the minute finger-like pores of the pristine membrane into enlarged finger-like cavities. The aforementioned channels exhibit interconnectivity with sponge-like structures and span vertically from the uppermost to the lowermost regions through the presence of a membrane cross-structure. It is noteworthy to mention that upon surpassing a PCLNPG content of 3 % in the casting solution, leading to increasing in its viscosity, accompanied by the migration of PCLNPG additives towards the lower layer. As a result, an observable outcome was the formation of a marginally expanded and more compact active layer in proximity to the lower surface of the PES membrane, as shown in Fig. 6A4. The observed phenomena can be due to the enhanced efficacy of the PCLNPG additives, resulting in heightened thermodynamic instability of the cast polymeric membrane. The observations presented align with the results reported by Farjami et al. [37]. The skin layer exhibits the crucial function of regulating permeability and retaining solutes, while the porous bulk layer primarily functions as a mechanical support structure [[38], [39], [40]].

PCLNPG introduction in the membrane induces variations in pore size and density, leading to two fundamental conclusions. (a) The dissolution of PCLNPG leading to viscosity elevation of the dope solution, consequently resulting in an increased concentration of polymer. (b) The thermodynamic stability of the polymer-doped solution was diminished, causing a delay in the demixing process during submerging in the coagulation basin. During this operation, the hydrophilicity of PCLNPG additives within the casting solution exerted an influence on the rate at which DMAc and water were exchanged. The observed phenomenon also exerted an influence on the rate at which precipitation occurred and the subsequent formation of the resulting structure within the membrane. Consistent results were observed in previous investigations [41,42].

**Fig. 7:**
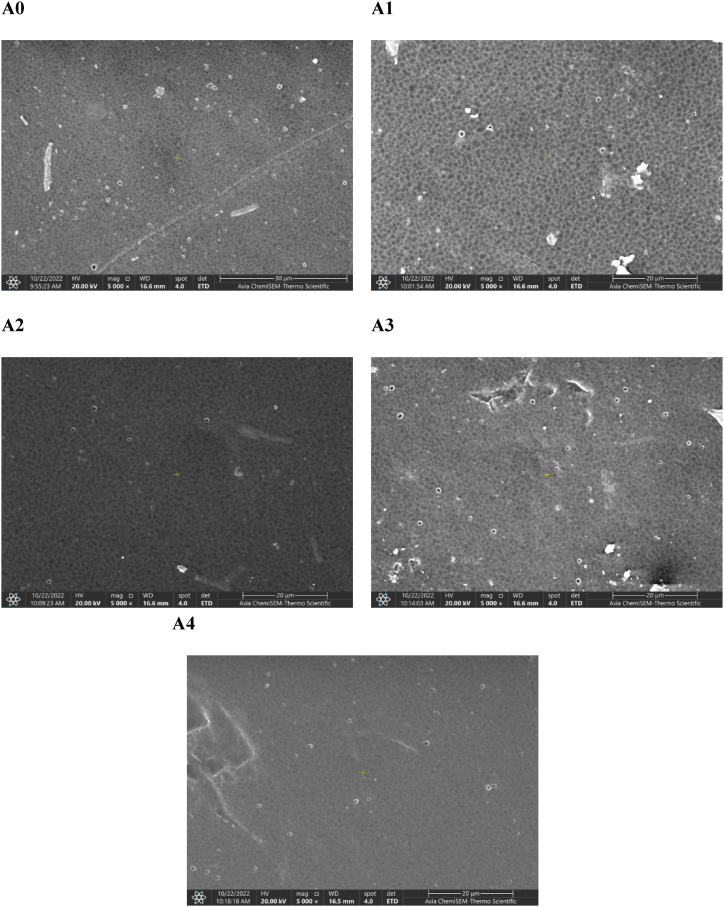
Top surface SEM images for PES/PCLNPG membrane A0, A1, A2, A3 and A4 with different wt% of PCLNPG additives of 0, 1, 2, 3, and 4 wt% respectively.

Fig. 7 depicts the SEM images of PES top surface created by varying PCLNPG amounts. By incorporating the PCLNPG additives into the casting solution, pores developed on the skin's surface (Fig. 7A1) in comparison with pristine membrane (Fig. 7A0).

**Fig. 8:**
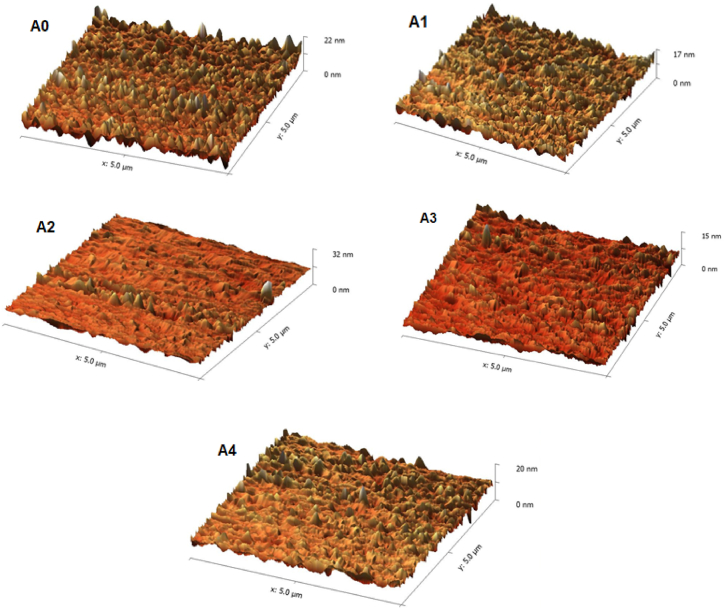
AFM images for PES/PCLNPG membrane A0, A1, A2, A3 and A4 with different wt% of PCLNPG additives of 0, 1, 2, 3, and 4 wt% respectively.

The density of the pore was significantly improved as a result of the polymeric solution's increased PCLNPG nano-polymer concentration (Fig. 7A2 and A3). Be aware that only very modest amounts of PCLNPG were required to begin pore development in the membrane skin layer which regulates membrane selectivity. Adding PCLNPG to the dope solution is anticipated to exert rigorous control over the membrane's functionality. The PCLNPG accumulation in the interface between water and casted membrane (during phase change) as well as its influence during coagulation process may be used to explain the increase in porosity with increasing in PCLNPG [22]. However, membrane's porosity slightly decreased when the PCLNPG concentration exceeded 3 wt% (Fig. 7A4). This could have happened as a result of the dope solutions' increased viscosity, which hindered membrane pore formation and growth by impeding the mobility of nano-polymer particles.

### 4.4 Atomic force microscopy (AFM)

AFM analysis was used to evaluate the surface topography of PES/PCLNPG membranes. For this purpose, 5 × 5 μm^2^ three dimensional AFM images were depicted in Fig. 8A0, A1, A2, A3 and A4 for PES/PCLNPG membrane with different wt% of PCLNPG additives of 0, 1, 2, 3, and 4 wt%, respectively. The highest points on membrane surface can be recognized by bright regions, while the valleys can be recognized by dark ones. Also, Table 2 displayed the surface roughness parametrs such as root mean squre height (Sq), maximum height (Sz) and Arithmetic mean height (Sa). As shown in Table 2, all the roughness parameters were reduced with increasing the addition of PCLNPG adittives and reach to minimum values at 3 wt% PCLNPG nanopolymer. In comparison with pristine PES, the roughness parameters Sq, Sa and Sz of membrane surface with 3 wt% of PCLNPG were reduced by 50, 55 and 51.5 % respectively. These results indicate that a smoother surface was attained and in turn the surface of membrane will has more resistance to the adsorption of foulants [43].

**Table 2:** The roughness parameters of the surface of PES/PCLNPG membranes.

Membrane Code	Sq (nm)	Sa (nm)	Sz (nm)
Aᴏ	29.41	25.08	151.3
A1	24.21	19.26	116.9
A2	32.77	28.23	128.2
A3	14.79	11.24	73.37
A4	32.78	27.88	132.7

### 4.5 Membranes contact angle (CA), porosity and thickness

**Fig. 9:**
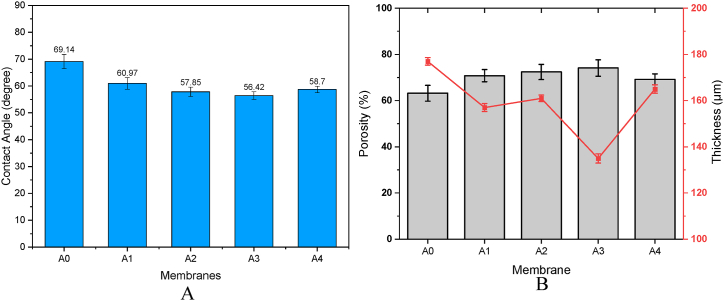
Effect of PCLNPG amount on (A) contact angle, (B) porosity and thickness.

CA measurements were carried out to assess the membrane hydrophilic properties. The consideration of hydrophilicity is of utmost importance in membrane applications due to its ability to facilitate fouling control and, consequently, enhance the longevity of the membrane [44]. The investigation focused on evaluating the surface hydrophilicity of membranes composed of pure PES and PES/PCLNPG. The outcomes of this analysis are visually depicted in Fig. 9A. The measured CA of the pure PES membrane was found to be 69.14°, a value that falls within the established range of contact angles commonly observed and documented for PES membranes in scientific literature. The magnitude of the contact angle (CA) exhibited a significant decrease following the introduction of a 1 wt percent of PCLNPG nano-polymer, ultimately stabilising at a value of 60.97°. With more increasing in PCLNPG loading, an observable reduction in CA was noticed, indicating a significant enhancement in the hydrophilic nature of the membrane surface. In addition, the water CA was quantified to be approximately 56.42° subsequent to the incorporation of 3 wt percent of PCLNPG nanopolymer. This observation signifies a noteworthy enhancement in the membrane hydrophilic properties. The observed enhancement can be due to the progressions in density of the pores, pore size, and the distribution of pore size, as will be elucidated subsequently. In the interim, when the concentration of PCLNPG was elevated to 4 wt percent (Wt.%), a slight augmentation in contact angle measurements was observed, reaching approximately 58.7°. The contact angle's value is contingent upon the intricate interplay of various parameters, including surface porosity, roughness, and pore size. This interplay may be influenced by the reduced porosity achieved due to the heightened in casting solution viscosity.

Also, Fig. 9B illustrates how the amount of PCLNPG additives affects the thickness and the porosity of the PES-PCLNPG membranes. With reference to pristine PES membrane, the porosity of PES membrane with 1 wt % PCLNPG additives increased from 63.2 % to 70.8 %. By rising the PCLNPG amount to 3 wt%, the membrane porosity reached 74.1 %. This was most likely due to the addition of hydrophilic additives, which enhanced membrane porosity. Manawi et al. [45] found that the hydrophilic additives have a considerable effect in improving the porous structure of membrane.

The use of PCLNPG additives, which make the coagulation process thermodynamically unstable, eliminated the thick top layer and increased the membrane porosity. Moreover, the voids in PES/PCLNPG membranes gradually changed morphology from microcavities to finger-like voids with narrow size distribution. Enhanced porosity leads to lower resistance to water flow through the mixed polymer membrane. However, the porosity of the produced membrane was diminished by increasing the PCLNPG loading (>3 wt%). This can be explained by the lower precipitation induced as the viscosity of the polymer dope solutions increases.

Also, Fig. 9B shows that increasing PCLNPG amount in casting solution to 1 and 2 wt% resulted in decreasing in membrane thickness from 177 to 157 and 161 μm, respectively. Whereas increasing the PCLNPG amount in casting solution to 3 wt% resulted in further decreasing in membrane thickness to 135 μm, while further increasing in the PCLNPG amount led to increment in membrane thickness of 165 μm. This behavior can explained taking into account the fast solvent/non-solvent exchange rate, hence, in a major tendency to decrease the membrane thickness. While increasing the PCLNPG amount resulted in increment polymeric casting viscosity, which in turn resulted in slight decreasing in solvent/non-solvent exchange rate, thus, increase the membrane thickness [[46], [47], [48]].

### 4.6 Pore size and distribution of pore size

**Fig. 10:**
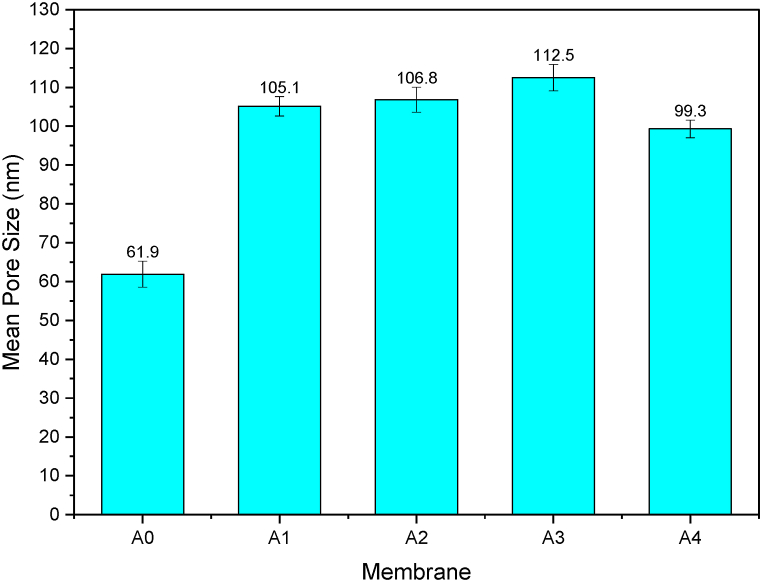
The change in PES/PCLNPG membrane pore size with various PCLNPG loadings.

**Fig. 11:**
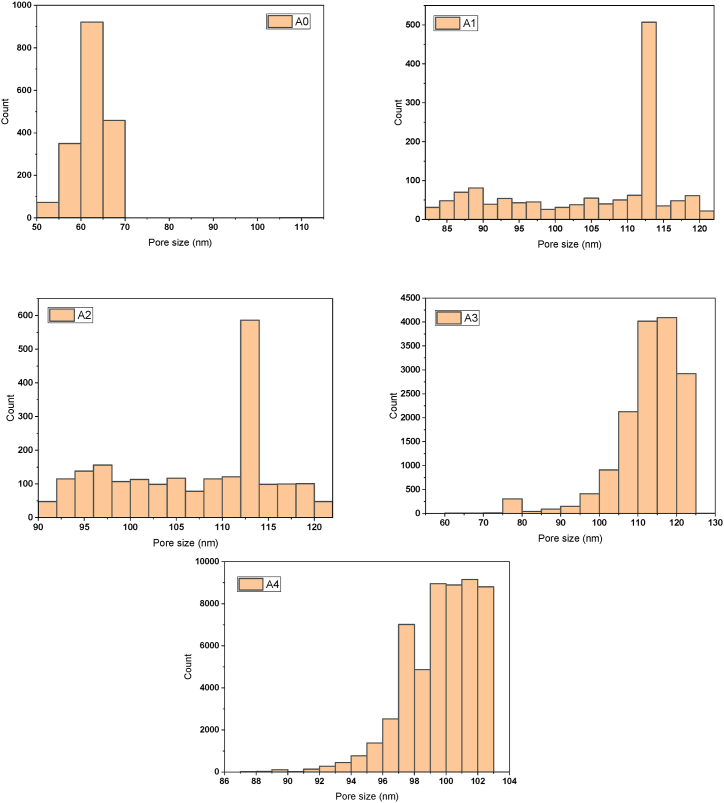
The change in PES/PCLNPG membrane pore size distribution with various PCLNPG loadings.

The most important factors for determining membrane selectivity and permeability are mean pore size and pore size distribution. The influence of PCLNPG content impregnation within the PES polymeric matrix on mean pore size was shown in Fig. 10. It is obvious to see that, there was a proportional increase in membrane pore size upon increasing the PCLNPG content. The pore size magnitudes were 61.9, 105.1, 106.8, 112.5, and 99.3 nm for membranes A0, A1, A2, A3, and A4, respectively. Moreover, Fig. 11 depicts the pore size distribution of PES/PCLNPG membranes. The frequency of pore size distribution for all PES/PCLNPG membranes is shifted to the right in reference to pristine PES membrane (Fig. 11A0). However, addition of 1 % and 2 % of PCLNPG (A1 and A2 membranes) in casting solution of PES resulted in wider the pore size distribution with slight increasing in pore density (Fig. 11A1 and A2). Further increasing in content of PCLNPG in PES solution up to 3 % and 4 %, narrow pore size distribution was obtained with 5-fold–10-fold of pore density higher than other membranes (Fig. 11A3 and A4). This result confirms the ultra-efficient impact of using PCLNPG additives as pore former. The rate at which the solution phase separates influences the pores size. As a matter of fact, the PCLNPG nano-polymer was the driving force behind the development of highly porous membranes. In contrast, the membrane pore size was reduced with rising of PCLNPG amount in casting solution more than 3 wt%. This can be explained by the lower precipitation induced as the viscosity of the PES-PCLNPG solutions increases. SEM images shown in Fig. 6, Fig. 7 support this phenomenon.

### 4.7 Performance of membrane

#### 4.7.1 Pure water flux (PWF)

**Fig. 12:**
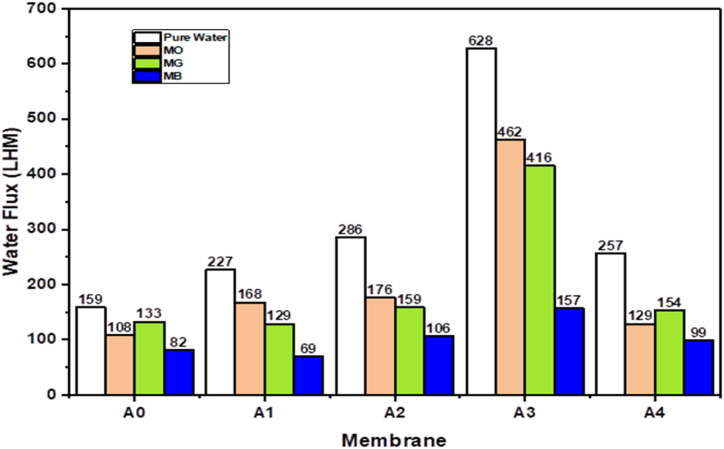
Pure water, methyl orange, malachite green and methylene blue dye solution flux for pristine PES and PES/PCLNPG membranes.

The membrane pore size and porosity is closely related with PWF and dye rejection, as previously documented [49]. Fig. 12 shows the PWF observed in PES/PCLNPG membranes with different compositions of nano-polymer PCLNPG. The experimental findings indicate a positive correlation between the concentration of PCLNPG nano-polymer in the casting solution and the PWF of the modified membrane, with a notable rise in PWF observed as the quantity of PCLNPG nano-polymer is augmented. The observed rise in performance can be ascribed to enhancements in hydrophilicity and pore density of membrane surface, porosity, and microvoids, along with the development of vertically interconnected finger-like pores that exhibit more desirable characteristics compared to the ones observed in the PES membrane [50]. The hydrophilicity exhibited by the surface of the membrane has the potential to enhance the attraction between the water molecules and membrane surface, thereby positively impacting the performance of the membrane that has been prepared, particularly in relation to its PWF [51]. Based on the empirical observations, it has been determined that the PWF of the polyethersulfone PES and PCLNPG composite membrane displayed a notable enhancement of 295 % in comparison with neat membrane. The utilisation of PCLNPG particulates led to the acceleration of DMAc/water exchange rate during the preparation of membrane and in result a membrane with enhanced porosity and larger cavities is formed. Consequently, the membrane's PWF and separation performance exhibited an increase in their values. Additionally, the introduction of PCLNPG nanoparticles induced a notable alteration in the membrane's morphology. Specifically, the pristine PES membrane, characterised by minuscule finger-shaped pores, underwent a transformation into larger pores integrated with macro voids in the PES/PCLNPG membrane. Consequently, this modification created a more streamlined pathway for water molecules to permeate, ultimately enhancing the PWF. The observed phenomenon indicates that the PWF of the polyethersulfone PES/PCLNPG membranes exhibited enhancement when the PCLNPG content increased by a maximum of 3 wt percent (wt%). This particular concentration was determined to be the optimal value based on the experimental results. Beyond this threshold, the PWF for the A4 membrane exhibited a slight decrease, presumably attributable to the elevated amount of PCLNPG in the casting solution at 4 wt %.

Unfortunately, when distilled water was replaced by dye solution, the membrane permeate flow was significantly reduced. Fig. 12 shows that the flux reduction for A0, A1, A2, A3 and A4 membranes was 32, 26, 38.5, 26.4, and 50 % for methyl orange dye, 16, 43, 44.4, 33.8, and 40 % for malachite green dye, and 48, 69.6, 63, 75, and % for methylene blue dye. This might be attributed to the adhesion of dye molecules on the surface of membrane, which constricts and blocks the pores [52]. Because of the higher concentration of PCLNPG particles, the modified membrane's surface became rougher and more susceptible to dye contamination [53]. Fig. 12 shows that the dye solution flow changes in all membranes followed a similar pattern to the PWFs. The ideal dye solution flux value was demonstrated by modifying the membrane with 3 wt% PCLNPG.

#### 4.7.2 Dye rejection

**Fig. 13:**
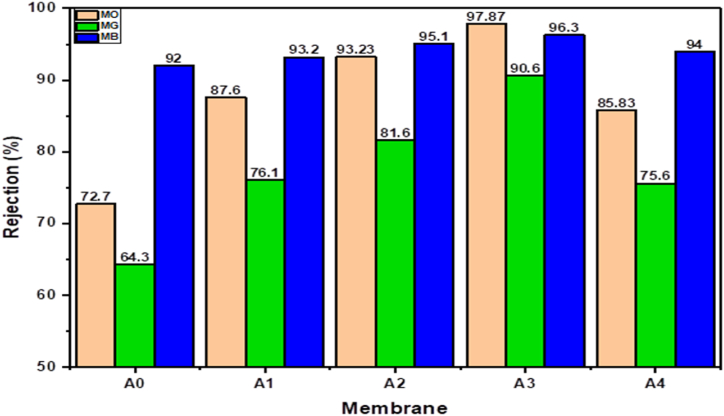
Rejection of pristine PES and PES/PCLNPG membranes against 30 ppm methyl orange, malachite green and methylene blue dye solutions.

Fig. 13 depicts the efficiency of removing (MO), (MG), and (MB) dyes from aqueous solutions using the prepared membranes with various PCLNPG additive compositions. As illustrated in Fig. 13, the removal percentages against all dyes solutions were found to increase with increasing PCLNPG wt.% up to 3 and then decreased at 4 wt%. The optimum dye rejection membrane was 3 wt%, with rejection ratios of 97.87, 90.6, and 96.3 % for MO, MG, and MB, respectively. The increase in dye rejection for PES/PCLNPG membranes could be assign to the impact of adding PCLNPG nano-polymer to the PES membrane, which makes the membrane surface more hydrophilic. This could have resulted in the dye molecules having lower affinity and interactions with the membrane surface, increasing membrane rejection. The decrease in dye rejection caused by the 4 wt% PCLNPG additive may be assign to a reduction in membrane hydrophilicity.

**Fig. 14:**
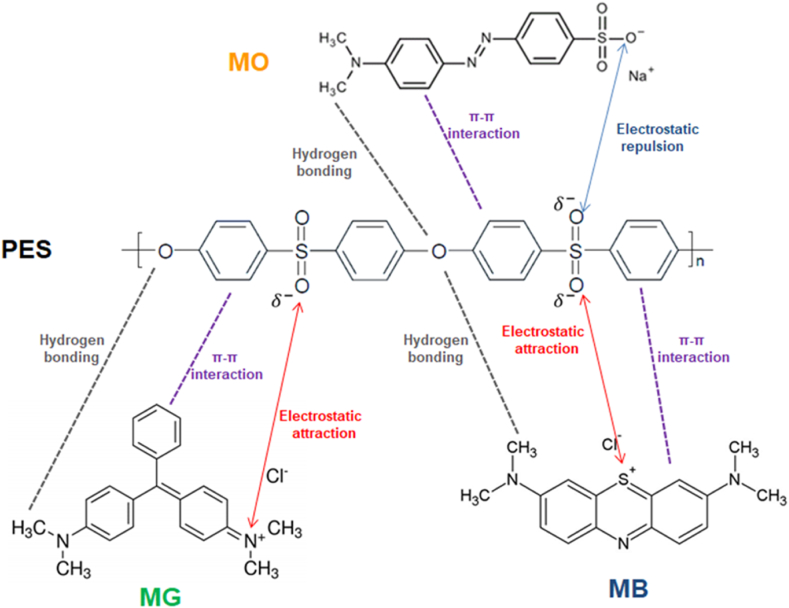
Proposed interaction mechanism between MO, MB and MG dyes with PES membrane.

Fig. 13 shows that dye rejection occurred in the order MO > MB > MG for all prepared membranes. These findings could be interpreted by the charge of dye molecules, which is meaningful in the ultrafiltration process. The higher rejection of anionic dyes like MO compared to cationic dyes like MB and MG is due to electrostatic charge repulsion, which results in weaker adhesion between membrane surface and dye molecules and higher water penetration [54]. The interaction mechanism of the MO, MB, and MG dyes with the PES membrane is shown in Fig. 14. The structure of dye molecules, in addition to the nature of the surface charge of the membrane and dye molecules, has an important function in the separation efficiency of the membranes [55]. As a result, MB was found to have a higher rejection rate than MG. This is primarily due to the linear structure of MB, as opposed to the spherical molecular shape of MG [56].

### 4.8 Comparison study

A comparative analysis was conducted to assess the attributes and efficacy of employing PCLNPG in the fabrication process of PES membrane. This investigation involved the juxtaposition of PCLNPG with several other pore formers that were previously documented in scientific literature. The aforementioned comparison was presented in Table 3, wherein the parameters of porosity, pore size, contact angle, PWF, and pollutant rejection were evaluated. The experimental results indicate that the newly developed PES/PCLNPG membranes exhibit a significantly elevated PWF and rejection efficiency in comparison to the prevailing membranes documented in the existing literature. According of this comparison, it can be inferred that the PES/PCLNPG membrane exhibits notable permeation and separation properties. Consequently, PCLNPG emerges as a promising choice for a pore former, enabling the modification of the morphological structure and subsequently enhancing membrane performance.

**Table 3:** Current work results versus the works presented in literature.

Membrane Material	Pore former	Porosity (%)	Mean Pore Size (nm)	Contact Angle (Degree)	PWF (LHM)	Rejection (%)	Mode of operation	Ref.
20 % PES	3 % PCLNPG	74.1	60.24	56.42	628	97.87 MO 90.6 MG 96.3 MB	UF	This work
20 % PES	5 % PTGM	81.21	54.91	52.58	203.1	93.8 BSA 95.6 SA	UF	[57]
20 % PES	4 % TGF	73.3	40.59	50.4	300	96 BSA	UF	[22]
15 % PES	0.119 % PMG	83	108.28	42.04	908	98 BSA	UF	[58]
15 % PVDF	0.6 kDa PEG	76	98	95	63	99 BSA	UF	[59]
PSf	5 % PEG (0.4–20 kDa)	59	6.88	_	77	78 BSA	UF	[60]
17 % PES	5 % PEG 400; 2 % Tween-20	35.31	73.2	_	36.9	93.3 BSA	UF	[61]
PES	0.4 kDa PEG (0–15) %	_	31.4	_	172	90 BSA	UF	[62]
15 % EPVC	2 % PEG1000 0.75 % boehmite	79.4	14.8	57	700	98 BSA	UF	[63]
14 % PAN	4 % PVP	55	_	76	100	92.47 % HA	UF	[64]
PES	3 % MOF	_	2.3	60	121.5	82.3 MB dye 98.6 CR dye	UF	[65]
18 % PES	2 % DES 1 % PVP	64	12.1	44.5	241.3	99 BSA	UF	[66]
15 % PSf	0.5%PVP/10%HP-β-CD	92	_	38.89	891	93 BSA	UF	[21]
17 % PES	10 % PVP	_	150	_	2439	90 HA	UF	[67]

## 5 Conclusions

In this study, a newly developed water-soluble polymeric nano-additive known as partially cross-linked nanoparticles graft copolymer (PCLNPG) was harnessed as a pore former in the fabrication of PES membrane for dye removal. The impacts of various ratios of PCLNPG (0-4 wt%) impregnation on the hydrophilicity, porosity, morphological structure, composition, and overall membrane performance were investigated. When made with a 3 wt% PCLNPG component, the modified membrane's porosity and hydrophilicity improved by 17 and 18 %, respectively, in comparison with pristine membrane. Besides that, the addition of PCLNPG material significantly alters the membrane structure. Increasing the PCLNPG content lead to form a membrane with porous structure.

Besides this, PWF and dye rejection was considerably improved, especially when the ratio of PCLNPG was 3 wt %. The membrane's pure water flux was 628 LMH, or approximately 3.95 times that of the pristine membrane, while the rejection of MG, MB, and MO dyes was 90.6, 96.3, and 97.87 %, respectively. According to these findings, the demonstrated performance characteristics of the PES/PCLNPG membrane make it a potentially advantageous option to treat the textile wastewater.

## Data availability statement

Data included in article/referenced in article.

## Additional information

No additional information is available for this paper.

## CRediT authorship contribution statement

**Kadhum M. Shabeeb:** Conceptualization, Data curation, Formal analysis, Investigation, Visualization, Writing – original draft. **Wallaa A. Noori:** Data curation, Formal analysis, Methodology, Visualization. **Ali A. Abdulridha:** Conceptualization, Data curation, Formal analysis, Investigation, Methodology, Validation, Visualization. **Hasan Sh Majdi:** Formal analysis, Methodology, Validation. **Mohammad N. Al-Baiati:** Conceptualization, Data curation, Formal analysis, Validation. **Ali A. Yahya:** Data curation, Formal analysis, Investigation, Methodology, Validation, Visualization, Writing – original draft. **Khalid T. Rashid:** Conceptualization, Data curation, Formal analysis, Investigation, Methodology, Visualization, Writing – original draft, Writing – review & editing. **Zoltán Németh:** Conceptualization, Formal analysis, Methodology, Validation, Writing – review & editing. **Klara Hernadi:** Conceptualization, Data curation, Formal analysis, Investigation, Methodology, Validation, Writing – review & editing. **Qusay F. Alsalhy:** Conceptualization, Data curation, Formal analysis, Investigation, Methodology, Resources, Software, Supervision, Validation, Visualization, Writing – original draft, Writing – review & editing.

## Declaration of competing interest

The authors declare that they have no known competing financial interests or personal relationships that could have appeared to influence the work reported in this paper.
